# The mechanism of Gejie Zhilao Pill in treating tuberculosis based on network pharmacology and molecular docking verification

**DOI:** 10.3389/fcimb.2024.1405627

**Published:** 2024-07-02

**Authors:** Yuhui Gao, Bingbing Shang, Yanyao He, Wen Deng, Liang Wang, Shaoguang Sui

**Affiliations:** ^1^ Emergency Department, The Second Affiliated Hospital, Dalian Medical University, Dalian, Liaoning, China; ^2^ Research and Teaching Department of Comparative Medicine, Dalian Medical University, Dalian, Liaoning, China

**Keywords:** Gejie Zhilao Pill, tuberculosis, traditional Chinese medicine, network pharmacology, molecular docking

## Abstract

**Introduction:**

Gejie Zhilao Pill (GJZLP), a traditional Chinese medicine formula is known for its unique therapeutic effects in treating pulmonary tuberculosis. The aim of this study is to further investigate its underlying mechanisms by utilizing network pharmacology and molecular docking techniques.

**Methods:**

Using TCMSP database the components, potential targets of GJZLP were identified. Animal-derived components were supplemented through the TCMID and BATMAN-TCM databases. Tuberculosis-related targets were collected from the TTD, OMIM, and GeneCards databases. The intersection target was imported into the String database to build the PPI network. The Metascape platform was employed to carry out Gene Ontology (GO) and Kyoto Encyclopedia of Genes and Genomes (KEGG) enrichment analysis. Heatmaps were generated through an online platform (https://www.bioinformatics.com.cn). Molecular docking was conducted between the core targets and core compounds to explore their binding strengths and patterns at the molecular level.

**Results:**

61 active ingredients and 118 therapeutic targets were identified. Quercetin, Luteolin, epigallocatechin gallate, and beta-sitosterol showed relatively high degrees in the network. IL6, TNF, JUN, TP53, IL1B, STAT3, AKT1, RELA, IFNG, and MAPK3 are important core targets. GO and KEGG revealed that the effects of GJZLP on tuberculosis mainly involve reactions to bacterial molecules, lipopolysaccharides, and cytokine stimulation. Key signaling pathways include TNF, IL-17, Toll-like receptor and C-type lectin receptor signaling. Molecular docking analysis demonstrated a robust binding affinity between the core compounds and the core proteins. Stigmasterol exhibited the lowest binding energy with AKT1, indicating the most stable binding interaction.

**Discussion:**

This study has delved into the efficacious components and molecular mechanisms of GJZLP in treating tuberculosis, thereby highlighting its potential as a promising therapeutic candidate for the treatment of tuberculosis.

## Graphical Abstract

A flowchart of this study on treating tuberculosis with Gejie Zhilao pills.
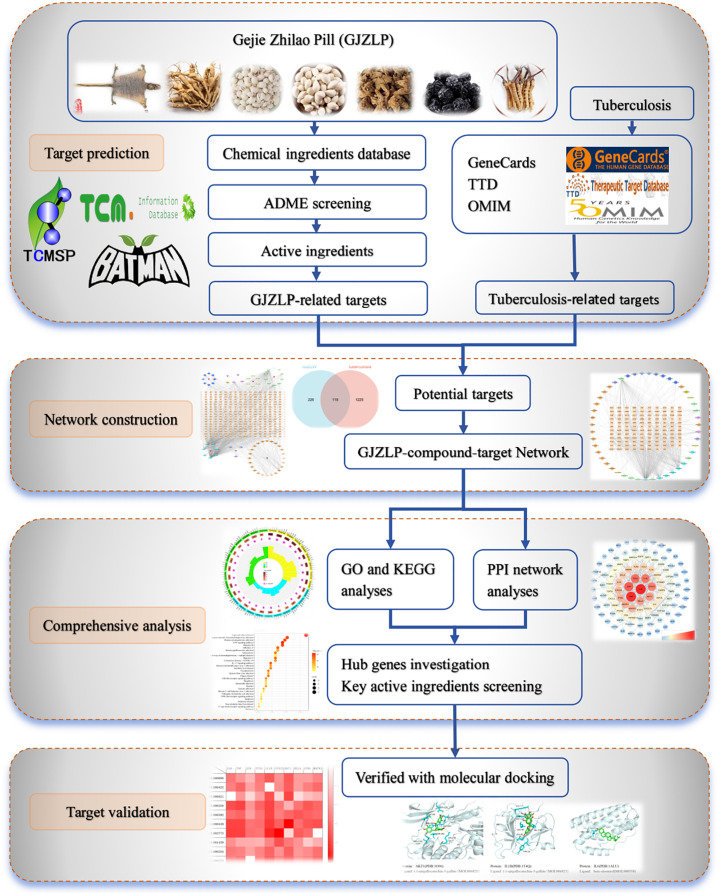

## 1 Introduction

Tuberculosis, a chronic infectious illness, results from the infection caused by Mycobacterium tuberculosis (Taye et al., 2021). It primarily affects the lungs and is known as pulmonary tuberculosis, but it can also involve various organs throughout the body (Ahmed et al., 2022). The clinical manifestations of tuberculosis include low-grade fever, night sweats, cough, sputum production, hemoptysis, anorexia, fatigue, weight loss, and more. Pulmonary tuberculosis is the most common type of tuberculosis accounting for the majority of total cases across various organs. Historically, tuberculosis has been widespread globally and once posed a significant threat to human health. However, with the continuous discovery of anti-tuberculous drugs, its prevalence has been partially controlled. Nevertheless, in recent years, due to factors such as neglect of tuberculosis by some countries, decreased financial investment, population growth, increasing migrant populations, and the spread of HIV, the decline in the prevalence of tuberculosis has been slow, and there has even been a resurgence in some countries and regions (Koegelenberg et al., 2021).

Currently, tuberculosis remains the primary cause of adult mortality due to infectious diseases globally (Furin et al., 2019). According to the World Health Organization (WHO), nearly one-fourth of the world’s population harbors latent infection with Mycobacterium tuberculosis (Suárez et al., 2019). Furthermore, over 10 million individuals are diagnosed with tuberculosis each year. Tuberculosis stands as the second leading killer of humans, trailing only behind HIV/AIDS. The disease is particularly prevalent among socially marginalized groups and communities with low socio-economic status (Natarajan et al., 2020). The 2023 Global Tuberculosis Report reveals that the number of new tuberculosis cases globally reached 10.6 million in 2022, marking a new historical peak. In such a context, the development of new anti-tuberculosis drugs is very important.

In China, traditional Chinese medicine (TCM) is increasingly being utilized in the treatment of tuberculosis (Wang et al., 2015). Studies have demonstrated that the therapeutic effect of combining TCM with Western medicine for tuberculosis treatment is superior to using Western medicine alone, which contributes to reducing the incidence of adverse reactions (Li et al., 2020). As a member of the treasure trove of TCM, the Gejie Zhilao Pill embodies the rich medical wisdom and cultural heritage of the Chinese nation. Over the course of long-term medical practice, GJZLP has gradually gained widespread recognition and application, becoming one of the significant TCM preparations for the treatment of tuberculosis and other related diseases. It is primarily used to nourish the lungs, suppressing coughs, and treating tuberculosis, and it is suitable for treating symptoms such as pulmonary tuberculosis, hot flashes, sweating at night, cough and hemoptysis. Among its primary constituents are gecko, stemona tuberosa, fritillaria ussuriensis, ginkgo biloba, bletilla striata, prunus mume, cordyceps sinensis, which work synergistically to exert therapeutic effects. Up to now, it is still widely used in clinical practice and plays a positive role in the treatment of tuberculosis. The Gejie Zhilao Pill is a large, brownish-colored honeyed pill with a subtle aroma and a sweet but slightly bitter taste. It is composed of natural ingredients, exhibiting minimal side effects and rare instances of severe adverse reactions. The multifaceted components of GJZLP target various aspects of the disease, demonstrating a synergistic effect and enhancing therapeutic outcomes. Compared to monotherapy, GJZLP intervenes more comprehensively in the pathological process of tuberculosis, thereby improving treatment efficacy. Furthermore, through rational compatibility and processing, the toxic and adverse effects of its medicinal components can be mitigated. GJZLP possesses antimicrobial, anti-inflammatory, antioxidant, immune-boosting, liver-protecting, BCG-potentiating, treatment-shortening, and prognosis-improving properties, conferring significant advantages in the treatment of tuberculosis. However, research on its application in the treatment of tuberculosis is still in its infancy, necessitating larger-scale, multicenter, randomized controlled trials to confirm its effectiveness and safety. GJZLP holds considerable potential in the treatment of tuberculosis. With the deepening of modern medical research, it is poised to become a widely used therapeutic agent for tuberculosis, ultimately improving the prognosis and quality of life for patients. Simultaneously, this development offers new research avenues and opportunities for the global application of TCM in the treatment of tuberculosis.

Overall, the Gejie Zhilao Pill is a traditional Chinese medicine compound that functions to nourish the lungs, suppress coughs, and treat tuberculosis, exhibiting good therapeutic effects on pulmonary tuberculosis and related symptoms. However, to date, there have been few studies on the mechanism of the Gejie Zhilao Pill in treating tuberculosis, and further research is urgently needed.

Network pharmacology is an emerging research paradigm that has superseded the traditional drug discovery model, which was predominantly focused on the “one drug, one target, one disease” approach. Instead, It has introduced an innovative research paradigm that delves into the intricate network interconnections between multiple targets and diverse diseases (Zhao et al., 2023). This methodology is particularly effective in discovering bioactive substances and elucidating the mechanistic actions of traditional Chinese medicine (TCM) prescriptions, thereby providing a novel research paradigm for the transition of TCM from empirical medicine to evidence-based medicine systems (Nogales et al., 2022). Network pharmacology has not only accelerated the discovery of TCM therapeutics but also improved existing drug discovery strategies. For instance, through similarity searches and identification of potential components in Chinese medicinal herbs, combined with GO enrichment analysis, pathway enrichment analysis, and network pharmacological analysis, it is possible to reveal the mechanistic actions and theoretical essences of a particular TCM formula (Jiashuo et al., 2022).

Molecular docking technology holds a pivotal position in the realm of computer-assisted drug design. It simulates the mutual recognition and interaction process between receptors and ligands by leveraging the principles of geometric spatial structure matching and chemical energy interactions between molecules (Pinzi and Rastelli, 2019). By predicting the three-dimensional specific structure and chemical characteristics of receptor molecules, as well as the interactions between receptor molecules and other ligand molecules, molecular docking technology can accurately predict their binding sites, modes of action, and affinities (Sahu et al., 2024). Furthermore, it enables the screening of ligand molecules and conformations that exhibit optimal affinity for the receptor molecule. This technique is widely used in drug discovery and drug repositioning, and it holds significant importance in screening effective components that truly exert pharmacological effects and elucidating the mechanisms of action of TCM in treating diseases (Stanzione et al., 2021).

Overall, both network pharmacology and molecular docking technology serve as crucial tools in modern drug research, each possessing unique advantages and application domains that collectively drive the advancement of drug discovery and design (Jiao et al., 2021). By integrating these two techniques, our aim is to gain a deeper understanding of the interactions between drugs and biological systems, thus accelerating the process of novel drug discovery and development.

To delve deeply into the therapeutic mechanism of GJZLP in treating tuberculosis, this study first employed the TCSMP, TCMID, and BATMAN-TCM databases to gather the bioactive components of GJZLP, converting the target IDs to UniProt database format. Then, ADMETlab2.0 was utilized for Predictive Toxicity Analysis of Drug Components. Tuberculosis-related targets were subsequently collected from the TTD, OMIM, and GeneCards databases. Next, utilizing Cytoscape 3.10.0, a compound-target network was constructed, while the ggplot2 package facilitated the creation of a Venn diagram. This analysis allowed for the further construction of an effective compound-therapeutic target network, enabling the screening of core compounds. The STRING database was leveraged to construct a protein-protein interaction (PPI) network and pinpoint core targets. The Metascape platform was utilized to conduct GO enrichment analysis and KEGG pathway enrichment analysis. Additionally, molecular docking simulations were conducted for the core targets and core compounds to validate their binding stability. The entire research process is illustrated in the **Graphical Abstract**.

## 2 Materials and methods

### 2.1 Screening the active ingredients and targets of GJZLP

The active components and their corresponding targets of GJZLP were retrieved from the TCMSP database (http://tcmspw.com/tcmsp.php) (Ru et al., 2014), which encompassed Chinese herbs such as stemona tuberosa, fritillaria ussuriensis, ginkgo biloba, bletilla striata, prunus mume and cordyceps sinensis. Due to the absence of information on the animal-derived Chinese medicine Gecko in the TCMSP database, we retrieved relevant data from the TCMID (http://www.bidd.group/TCMID/) database (Huang et al., 2018) and the BATMAN-TCM (http://bionet.ncpsb.org/batman-tcm/) database (Liu et al., 2016). To ensure consistent screening criteria, all the acquired effective components were inputted into the TCMSP database for screening based on the criteria of oral bioavailability (OB) ≥ 30% and drug-likeness (DL) ≥ 0.18 (Xu et al., 2012). Subsequently, the corresponding targets of the filtered effective components of GJZLP were collected, and the target IDs were converted to the UniProt database format for further analysis. ADMETlab2.0 (https://admetmesh.scbdd.com) is an online platform for predicting pharmacokinetic and toxicity properties of drugs (Xiong et al., 2021), encompassing the characteristics of absorption, distribution, metabolism, excretion, and toxicity of the compounds (Tian et al., 2015). To analyze the major chemical components of GJZLP using ADMETlab2.0, we retrieved the SMILES identifiers of the primary chemical constituents from the PubChem database (Wang et al., 2014) and imported them into ADMETlab2.0 to obtain toxicity-related information.

### 2.2 Construct a compound-target network

Cytoscape is an open-source software project designed to integrate biomolecular interaction networks with high-throughput expression data and other molecular states into a unified conceptual framework (Shannon et al., 2003). The obtained drug component data and target data were imported into Cytoscape3.10.0 to construct the GJZLP-compound-target network. In the network, each component or target is represented by a node, and the relationship between the component and the target is represented by a connecting line.

### 2.3 Identification of predicted targets of tuberculosis

To gather tuberculosis-related targets, we searched the databases of GeneCards (https://www.genecards.org/) (Stelzer et al., 2016), OMIM (https://omim.org/) (Amberger et al., 2019), and TTD (http://db.idrblab.net/ttd/) (Zhou et al., 2024). We entered the keyword “tuberculosis” to retrieve disease-related targets from each database. After merging the relevant targets from the three databases, duplicate entries were removed.

### 2.4 Construction of PPI network

We compiled a list of drug targets and disease-related targets and used the ggplot2 package to create a Venn diagram (Jia et al., 2021). Overlapping targets were identified as therapeutic targets. Subsequently, a network diagram of effective component-therapeutic target was constructed. The information of therapeutic targets was imported into the STRING database (https://string-db.org/) (Szklarczyk et al., 2023), with the biological species set to “Homo sapiens” and the minimum interaction score set to “highest confidence (0.9)” (Szklarczyk et al., 2019). Isolated targets were hidden to obtain protein-protein interaction (PPI) network information (Han et al., 2020). The PPI data was then imported into Cytoscape3.10.0 for further visualization. Network analysis using Cytoscape was conducted to calculate the degree, BC, and CC of each node in the network diagram. These parameters allowed for a deep analysis of the relevance and importance of nodes within the interaction network. We ranked the targets based on their degree values and identified the top ten targets as potential core targets.

### 2.5 GO enrichment and KEGG pathway analysis

To investigate the biological functions involved in tuberculosis with respect to therapeutic targets, these targets were imported into the Metascape platform for GO and KEGG analysis (Zhou et al., 2019). GO analysis was utilized to screen biological processes (BP), cellular components (CC), and molecular functions (MF) (Aleksander et al., 2023). Additionally, KEGG enrichment analysis was employed to identify crucial signaling pathways involved in the biological processes (Kanehisa et al., 2017). The data obtained from GO enrichment analysis and KEGG pathway enrichment analysis were uploaded to the online platform for data analysis and visualization (https://www.bioinformatics.com.cn) for further visualization processing.

### 2.6 Molecular docking

The three-dimensional structures of the core components of GJZLP were downloaded from the PubChem database (https://pubchem.ncbi.nlm.nih.gov/) and converted to the “PDB” format using PyMOL software. Each chemical component was appropriately modified to calculate charges, select torsions, and set as ligands. Core proteins corresponding to tuberculosis-related genes were searched in the Protein Data Bank (https://www.rcsb.org/). After eliminating free water molecules by adding all hydrogen atoms and calculating charges, the proteins were set as macromolecules using AutoDockTools (Goodsell et al., 2021). We selected a ligand and a macromolecule for each molecular docking, and generated “dlg” files with corresponding Grid Box settings based on protein size. Conformations were selected based on the optimal affinity and the most stable intermolecular forces, and a heatmap was plotted accordingly. The three-dimensional structures were exported as “PDBQT” files and visualized using PyMOL (Seeliger and de Groot, 2010).

## 3 Results

### 3.1 Compound-target network and analysis

**Figure 1:**
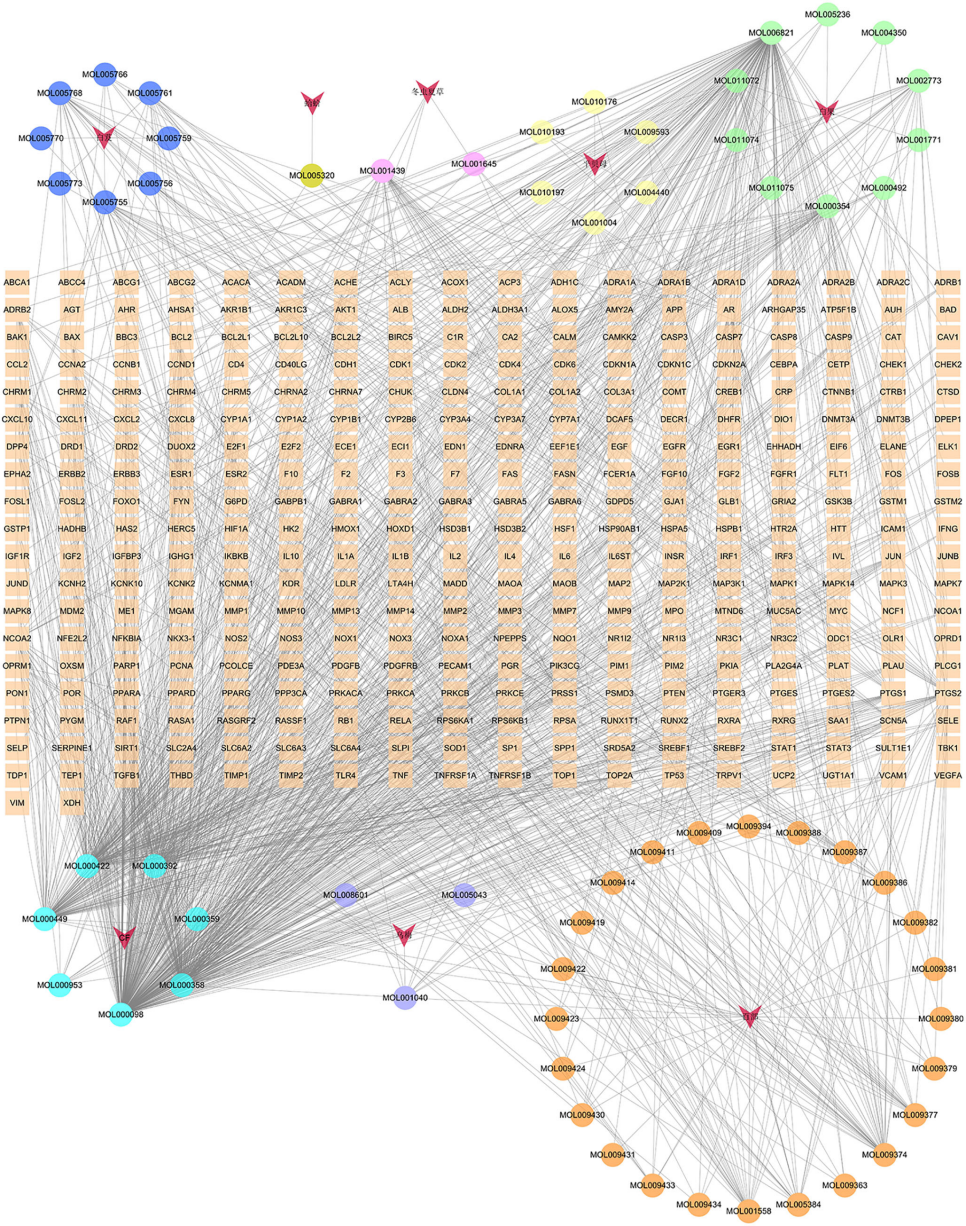
Herbal medicine-compound-target network of GJZLP (The circle represents the component, The V shape represents the traditional Chinese medicine, and the rectangle in the middle represents the target OF GJZLP).

In the GJZLP-compound-target network, there are 413 nodes and 1410 edges, comprising 61 chemical components and 344 targets, as depicted in **Figure 1**. The effective components of the six Chinese herbal medicines in GJZLP were retrieved from the TCMSP database. Specifiaally, 28 components were sourced from Stemona tuberosa, 7 from Fritillaria ussuriensis, 15 from Ginkgo biloba, 8 from Bletilla striata, 8 from Prunus mume, and 4 from Cordyceps sinensis. Among them, 7 components were present in multiple herbal medicines. Due to the lack of information on animal-derived Chinese medicines in the TCMSP database, we retrieved and supplemented the effective components of Gecko from the TCMID and BATMAN-TCM databases. To ensure consistent screening criteria, the effective components of gecko were screened from TCMSP database by uniform standards, thus identifying one active ingredient.

### 3.2 Effective compounds-therapeutic target network and analysis

**Figure 2:**
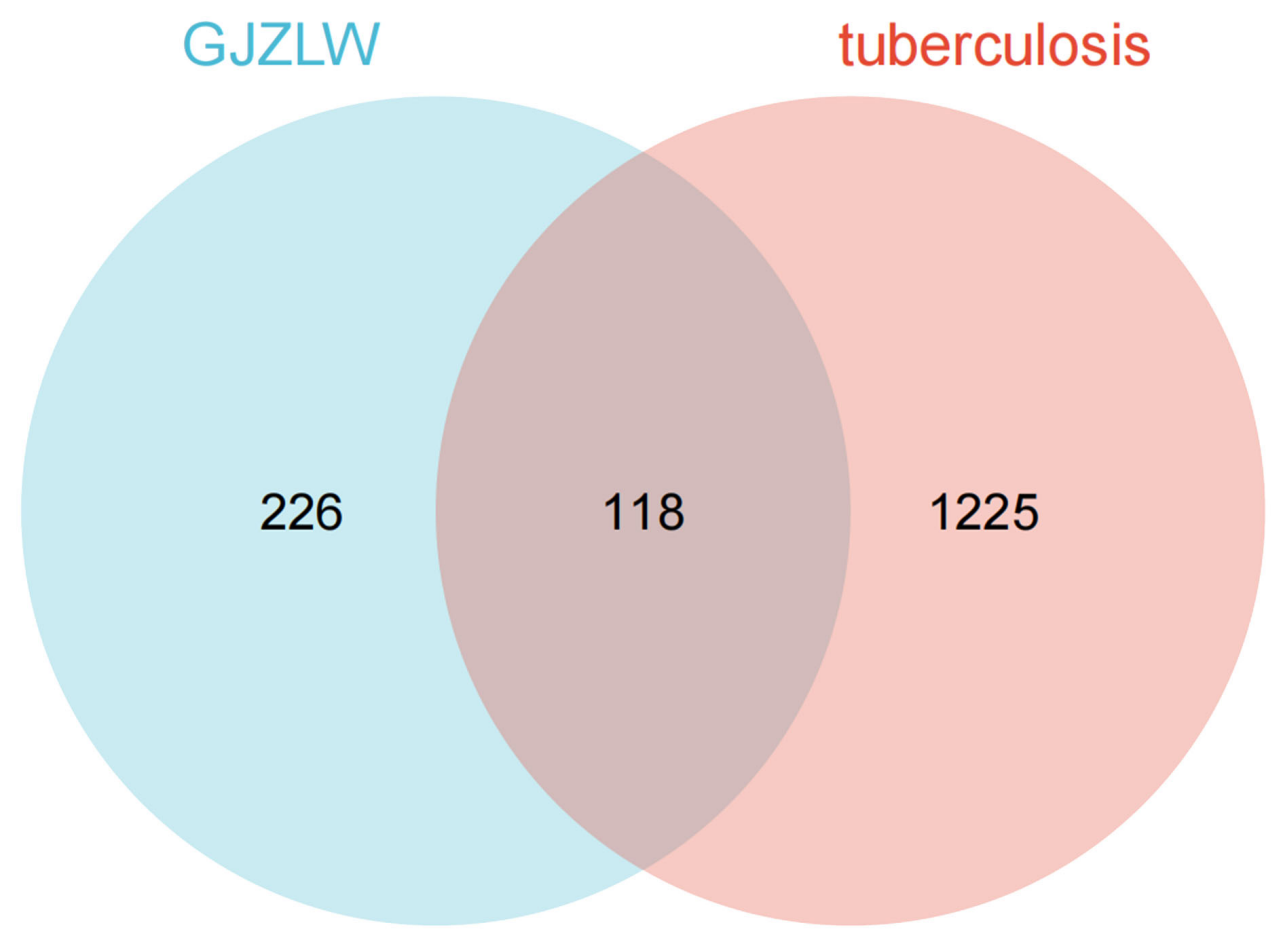
Venn diagram of the target of GJZLP and the target of tuberculosis.

**Figure 3:**
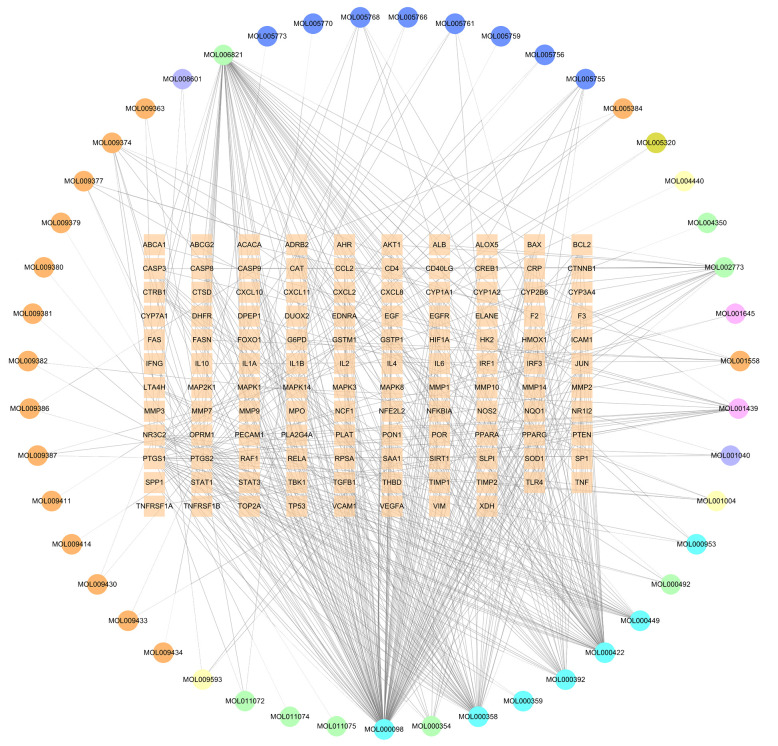
Effective compound-therapeutic target network (The circle represents the effective component, and the square in the middle represents the therapeutic target).

After obtaining the tuberculosis-related targets, the Venn diagram revealed 118 common targets shared between the effective components of GJZLP and tuberculosis-related targets (**Figure 2**). These targets have been identified as therapeutic targets. In the further constructed network of effective compounds-therapeutic targets based on these therapeutic targets, there were 165 nodes and 503 edges, comprising 47 effective compounds and 118 therapeutic targets (**Figure 3**). The top 10 compounds were selected as core compounds based on their Degree, BC, and CC values, as shown in **Table 1**.

**Table 1:** Top ten compounds information of GJZLP network.

MOL ID	Compound	Molecule structure	Degree	OB	DL	Average Shortest Path Length	Betweenness Centrality	Closeness Centrality	Hepatotoxicity	AMES Toxicity	Herb
MOL000098	quercetin	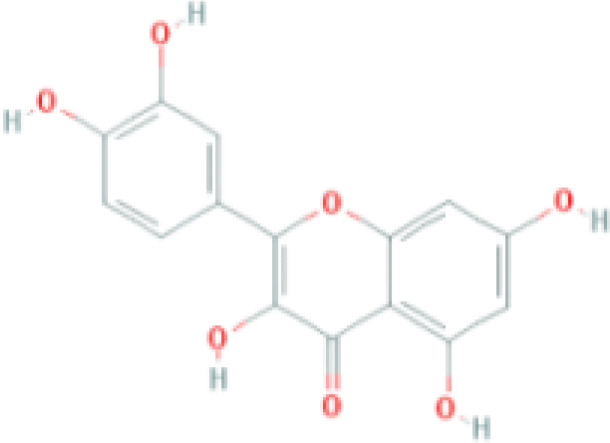	150	46.43	0.28	1.914634146	0.454008164	0.522292994	**—**	**+**	Ginkgo biloba Prunus mume
MOL000422	luteolin	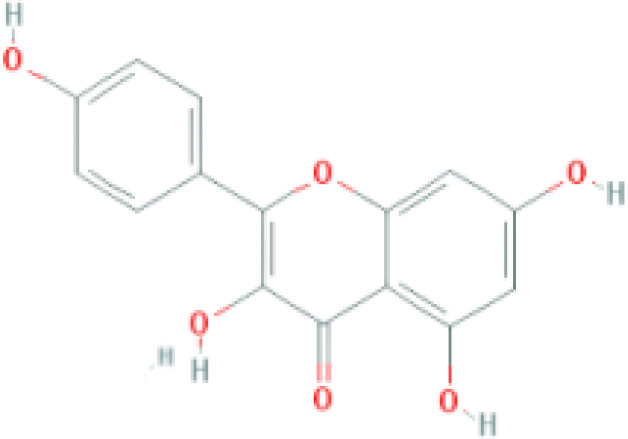	60	41.88	0.24	2.451219512	0.078896442	0.407960199	**—**	**+**	Ginkgo biloba Prunus mume
MOL006821	Epigallocatechin Gallate	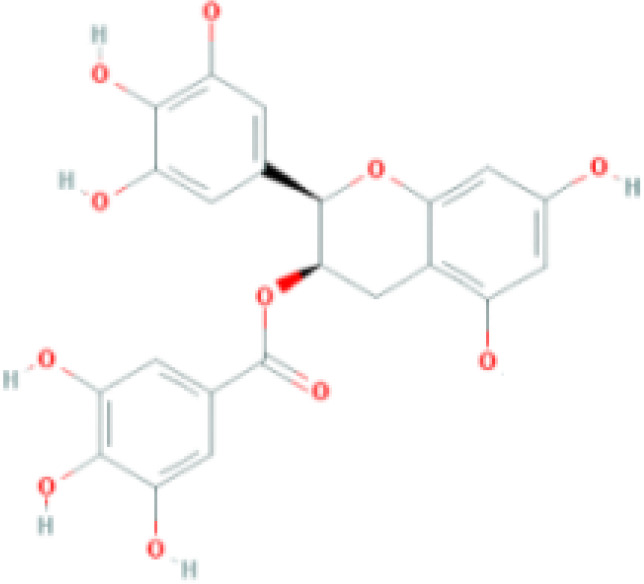	57	55.09	0.77	2.146341463	0.333159226	0.465909091	**–**	**–**	Ginkgo biloba
MOL000358	beta-sitosterol	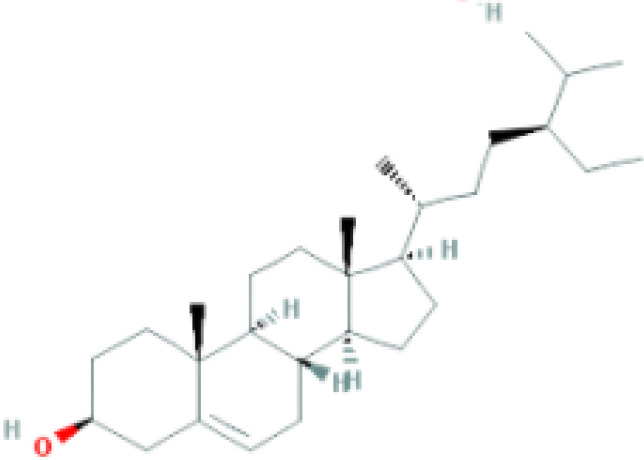	52	36.91	0.75	2.658536585	0.023859362	0.376146789	**–**	**—**	Stemona tuberosa Ginkgo biloba Prunus mume
MOL000392	formononetin	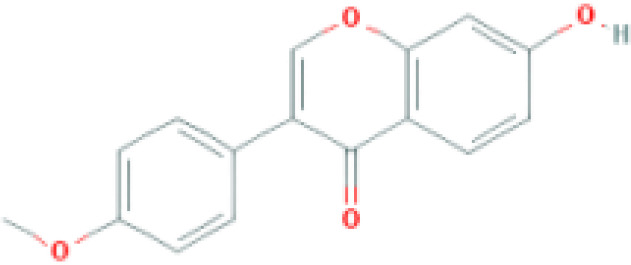	24	69.67	0.21	2.719512195	0.035755506	0.367713004	**—**	**–**	Stemona tuberosa Ginkgo biloba
MOL000449	Stigmasterol	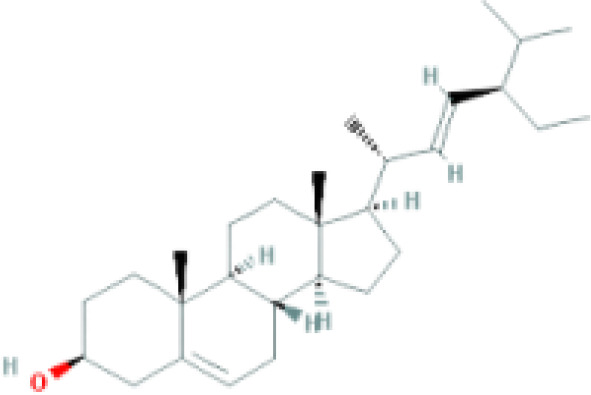	18	43.83	0.76	2.682926829	0.038300743	0.372727273	**—**	**—**	Stemona tuberosa Ginkgo biloba Prunus mume
MOL002773	beta-carotene	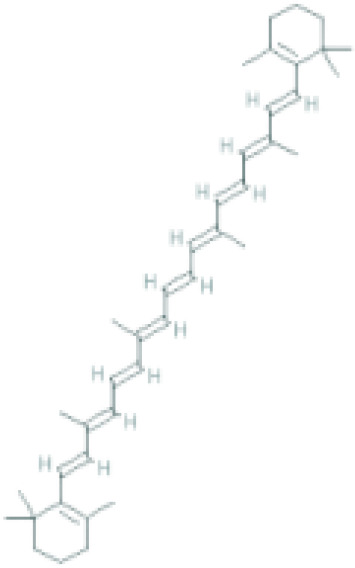	17	37.18	0.58	2.634146341	0.049200908	0.37962963	**–**	**–**	Ginkgo biloba
MOL001439	arachidonic acid	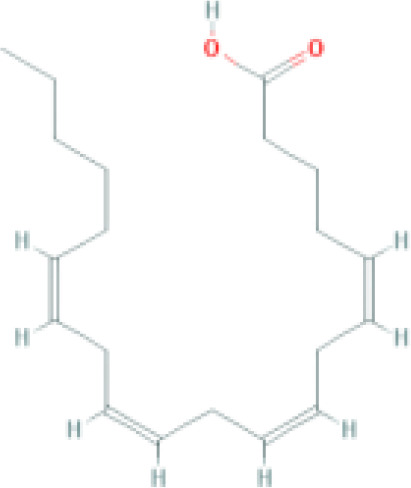	15	45.57	0.20	2.658536585	0.07091199	0.376146789	**—**	**-**	Cordyceps sinensis
MOL000354	isorhamnetin	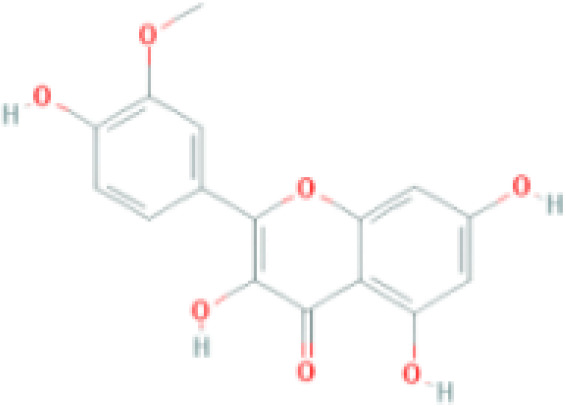	10	49.6	0.31	2.719512195	0.010605981	0.367713004	**—**	**+**	Ginkgo biloba
MOL009374	7-methoxy-3-methyl-2,5-dihydroxy-9,10-dihydrophenanthrene	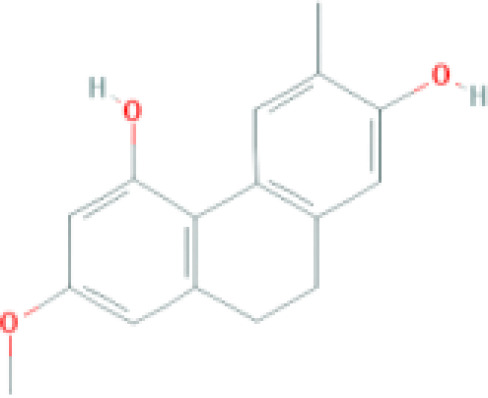	8	59.00	0.21	2.743902439	0.013785809	0.364444444	**–**	**+**	Stemona tuberosa

Tip: For the classification endpoints, the prediction probability values are transformed into six symbols: 0-0.1(—), 0.1-0.3(–), 0.3-0.5(-), 0.5-0.7(+), 0.7-0.9(++), and 0.9-1.0(+++). OB, Oral Bioavailability; DL, Drug-Likeness; AMES, The bacterial reverse mutation test. AMES Toxicity will only be shown as "++" or "+++" if the toxicity of the chemical is high. The absence of "++" and "+++" in the table proves that the toxicity of the TCM ingredients is low.

### 3.3 PPI network construction and analysis

**Figure 4:**
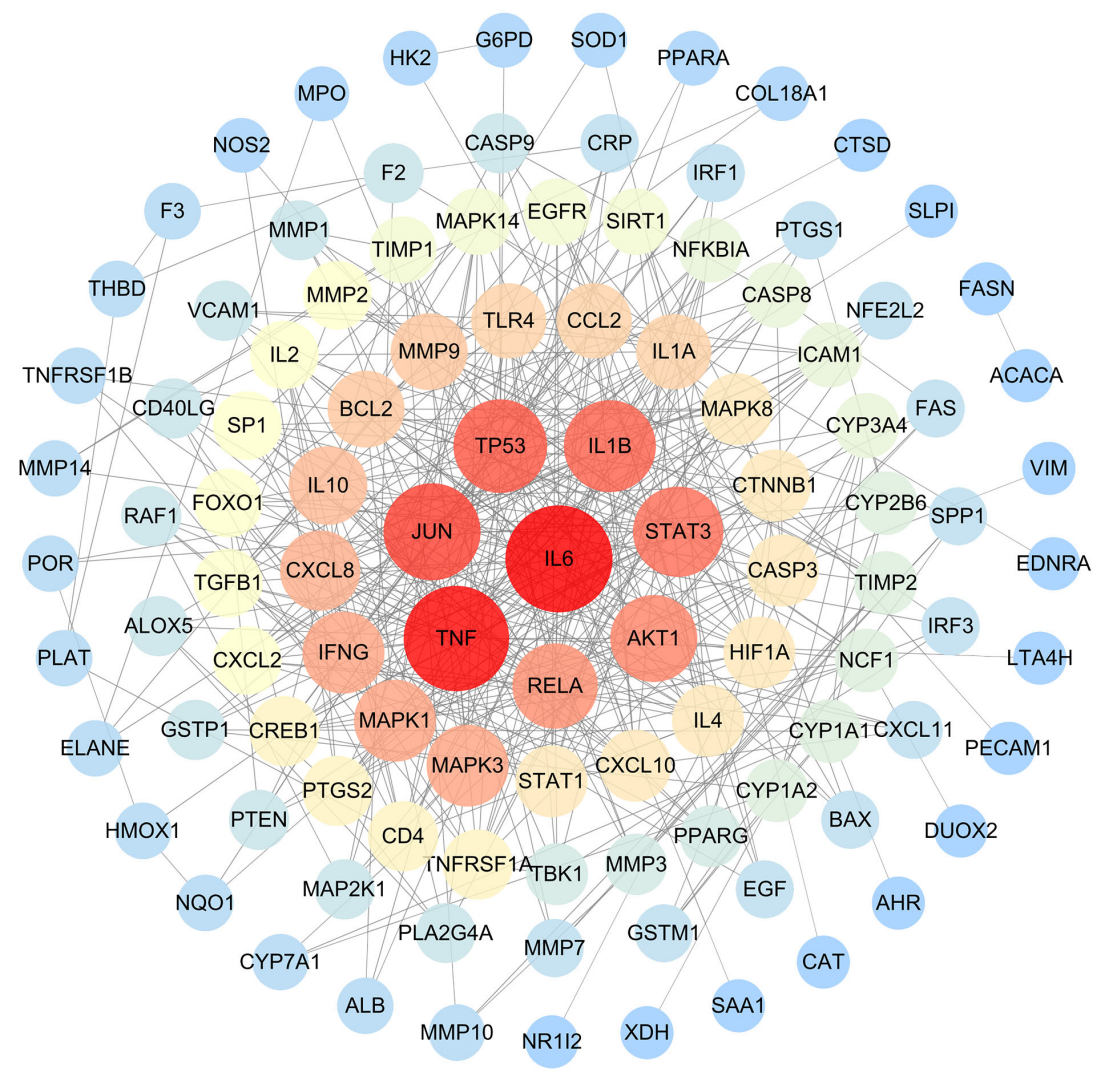
The PPI network of GJZLP and tuberculosis targets. Nodes represent proteins (The larger and redder a node is, the more important the protein it represents). Edge represents protein-protein association.

Using the therapeutic targets, we constructed a PPI network in the STING database. After removing outlier targets, a total of 107 target proteins were included in the study and visualized using Cytoscape3.10.0. In the visualization, nodes with higher degree values appeared larger and redder, indicating a greater likelihood of them being core targets (**Figure 4**). Based on the Degree, BC, and CC values, we selected the top 10 proteins as core targets, as shown in **Table 2**.

**Table 2:** Top ten targets information of PPI network.

Name	Degree	Betweenness Centrality	Closeness Centrality
IL6	33	0.192103259	0.502415459
TNF	32	0.147881096	0.497607656
JUN	27	0.1453186	0.479262673
TP53	25	0.095893042	0.426229508
IL1B	24	0.048365205	0.468468468
STAT3	23	0.081376202	0.468468468
AKT1	21	0.055590002	0.446351931
RELA	20	0.038235293	0.433333333
IFNG	18	0.008725639	0.393939394
MAPK3	18	0.037181845	0.448275862

### 3.4 GO enrichment and KEGG pathway analysis

**Figure 5:**
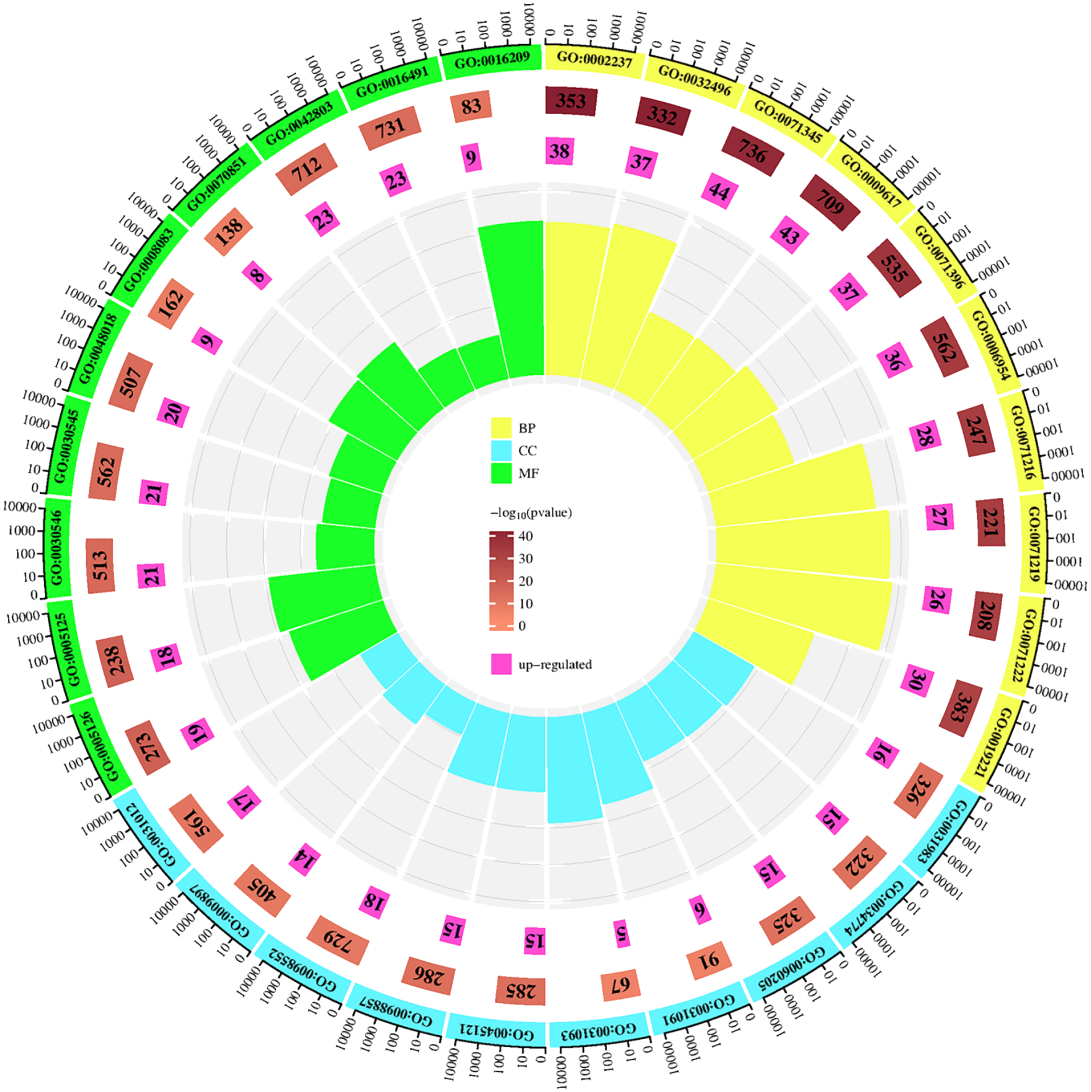
Top 10 GO terms of hub genes.

**Figure 6:**
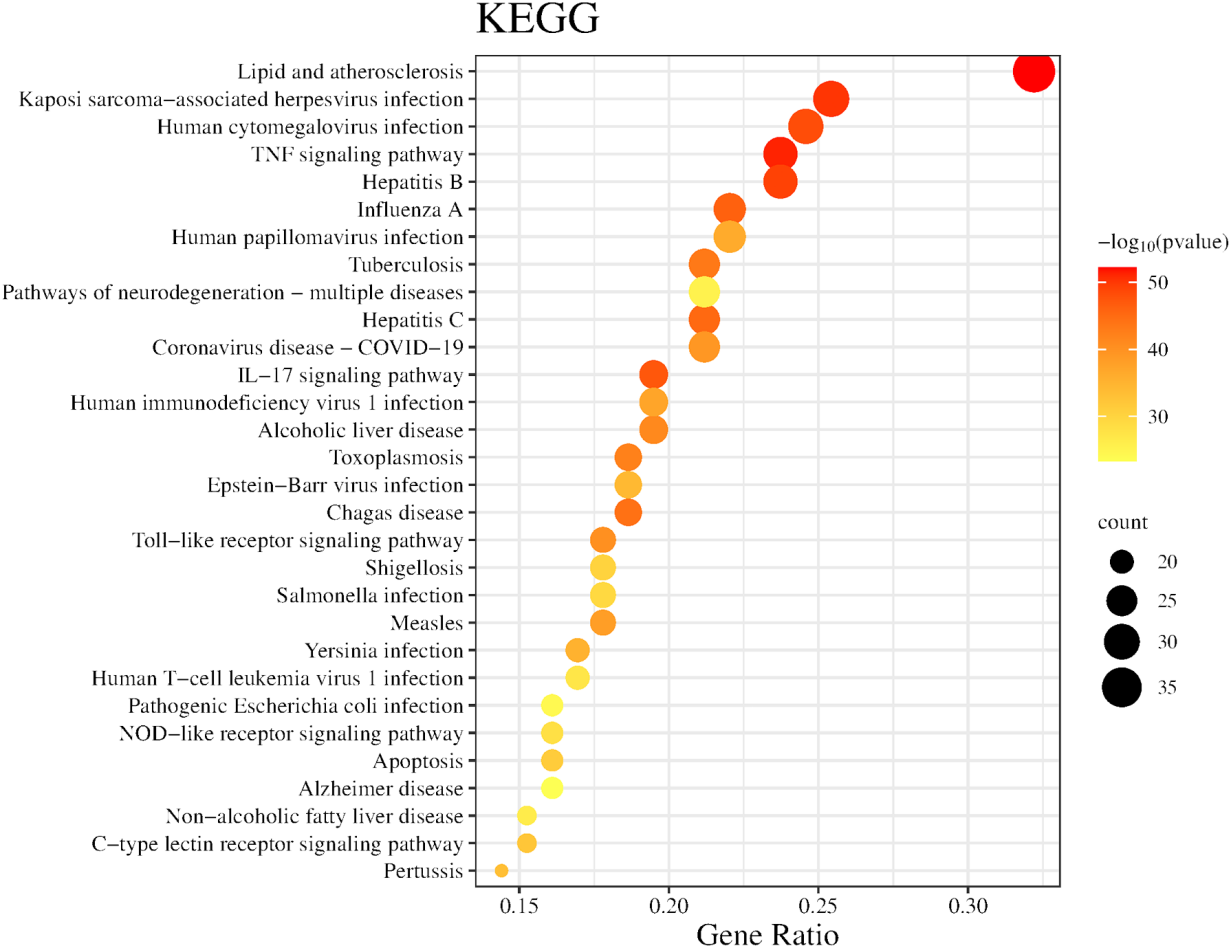
Top 30 KEGG pathway of hub genes.

In the GO analysis, for the purpose of visualization, we chose the ten most prominent biological processes, cellular components, and molecular functions, as depicted in **Figure 5**. These include biological processes such as response to molecules of bacterial origin, response to lipopolysaccharide and cellular response to cytokine stimulus. The major cellular components involved are vesicle lumen, secretory granule lumen, and cytoplasmic vesicle lumen. The primary molecular functions involved are cytokine receptor binding, cytokine activity, and signaling receptor activator activity. Utilizing KEGG enrichment analysis, we conducted a thorough investigation into the signaling pathway mechanisms of GJZLP in the treatment of tuberculosis, and visualized the top 30 ranked signaling pathways as depicted in **Figure 6**. The main signaling pathways implicated are Lipid and atherosclerosis, TNF, IL-17, Toll-like receptor and C-type lectin receptor signaling pathway.

### 3.5 Molecular docking study

**Figure 7:**
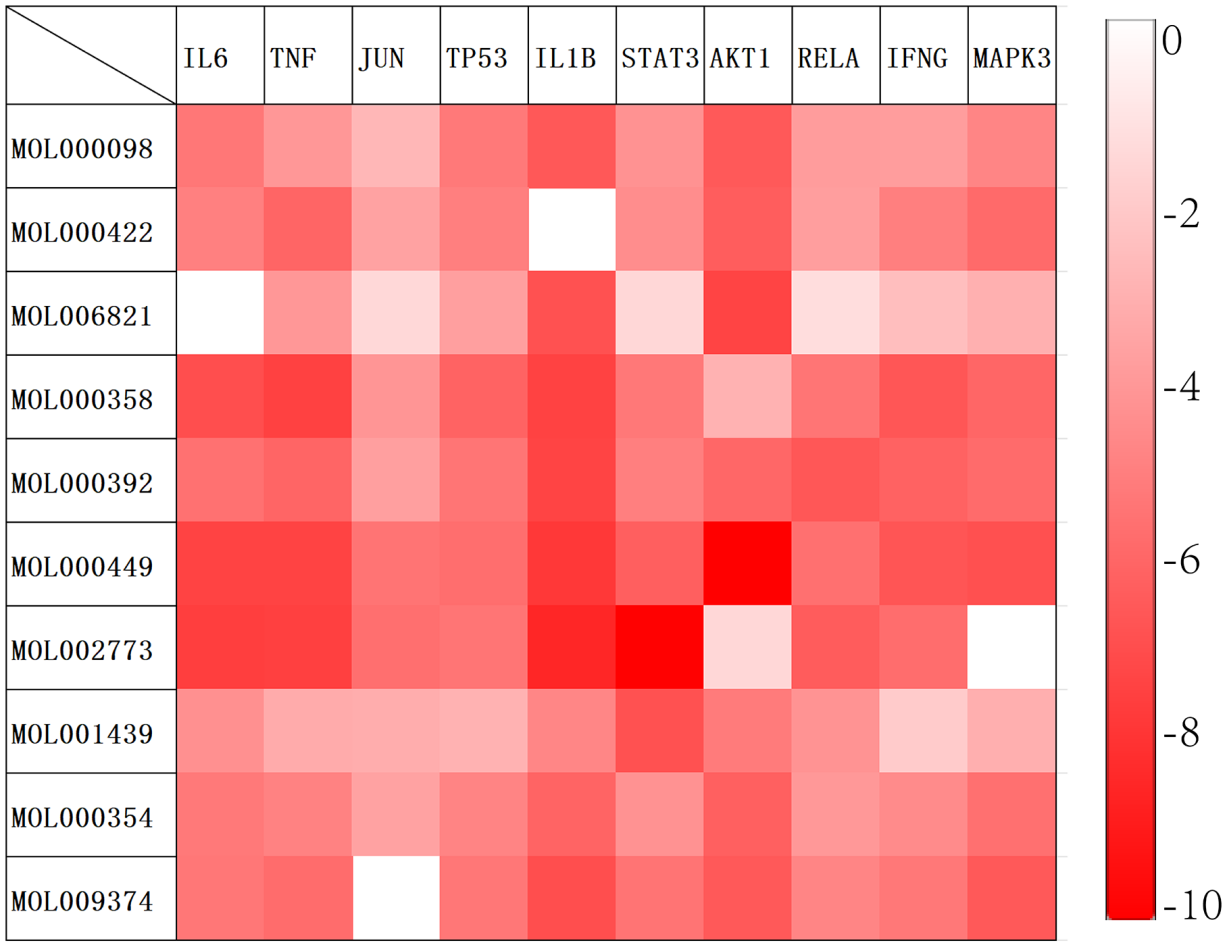
Molecular docking heat map (The redder the corresponding grid, the lower the binding energy and the more stable the binding).

**Figure 8:**
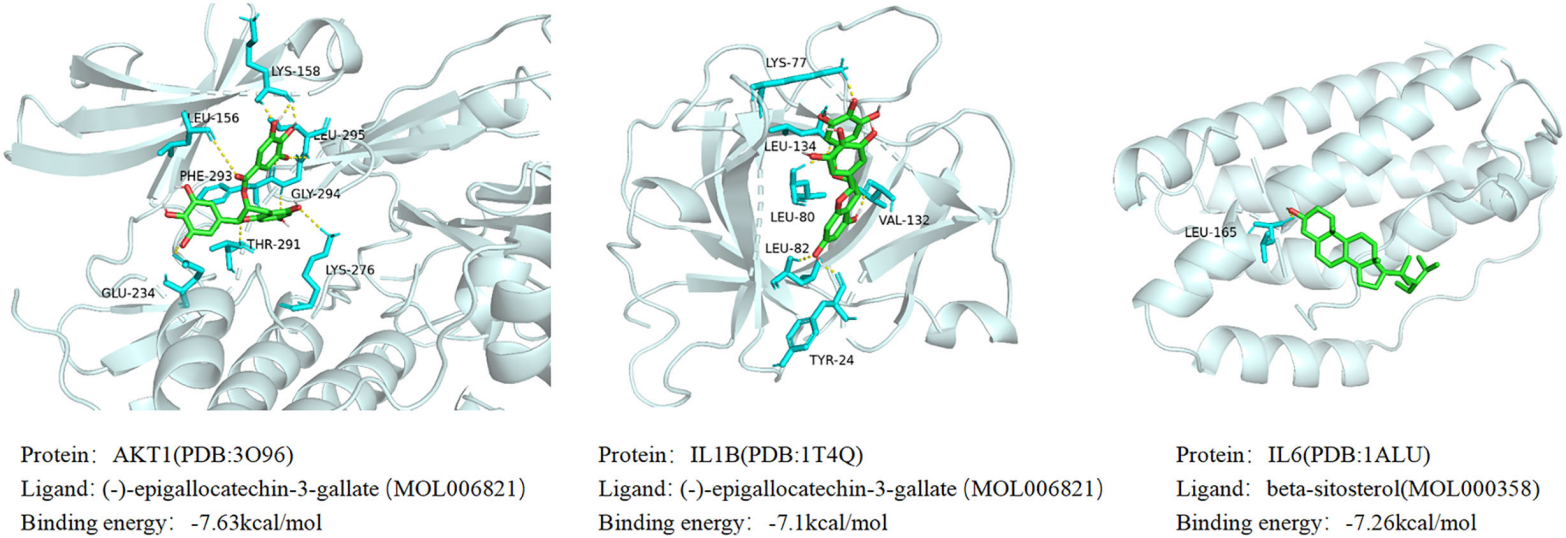
Molecular docking results of main chemical components of GJZLP.

Based on the results of the aforementioned network pharmacological analysis, we screened 10 core components of GJZLP and 10 core proteins associated with tuberculosis-related genes for molecular docking. This further corroborated the potential crucial role of these compounds in the treatment of tuberculosis. After docking using Autodocktools, we analyzed the docking results of proteins and ligands separately and plotted a heatmap based on the binding energies, as shown in **Figure 7**. Additionally, three representative models were selected for visualization, as depicted in **Figure 8**. A lower intermolecular binding energy indicates a higher binding affinity between molecules. Generally, a binding energy lower than -1.2 kcal/mol is considered to indicate good binding activity between molecules.

## 4 Discussion

The objective of this study is to explore the potential therapeutic mechanism of GJZLP in treating tuberculosis, leveraging network pharmacology and molecular docking approaches. Using databases such as TCMSP, TCMID, and BATMAN-TCM, a total of 61 chemical components and 344 targets were collected, and the connections between these components and targets were visualized through a network diagram. The relevant targets for tuberculosis were obtained from databases like TTD, GeneCards and OMIM. A Venn diagram revealed that there are 118 potential therapeutic targets for GJZLP in the treatment of tuberculosis. We further constructed a network diagram of effective components and therapeutic targets, and analyzed the core components, which mainly included quercetin, luteolin, epigallocatechin gallate, beta-sitosterol, formononetin, stigmasterol, beta-carotene, arachidonic acid, and isorhamnetin. According to the prediction of ADMETlab 2.0, these ingredients have low toxicity. These major chemical components, along with other effective components, can act on multiple targets in the network to produce therapeutic effects. Among the predicted main components, quercetin, luteolin, epigallocatechin gallate, and beta-sitosterol, which rank highly, have been proven to have good therapeutic effects on tuberculosis.

Quercetin can combat persistent tuberculosis infection by inhibiting isocitrate lyase of Mycobacterium tuberculosis (Shukla et al., 2015). The combined administration of quercetin and polyvinylpyrrolidone with antituberculous drugs can rapidly promote the fibrosis of epithelioid cell tubercles, leading to the separation of tuberculous granulomas through connective tissue and the emergence of a significant number of Langerhans cells and lymphocytes (Butova et al., 2016). Additionally, quercetin can prevent liver injury caused by antituberculous drugs through the activation of NRF2 and the blockade of NF-κB/TLR-4 (Sanjay et al., 2021).

Luteolin is a plant-derived liver protective immunomodulator that can promote anti-tuberculous immunity, shorten the treatment time of tuberculosis, and prevent disease recurrence when administered as a potential host-directed therapy together with isoniazid. Additionally, luteolin enhances the activity of natural killer cells and natural killer T cells, both of which possess anti-tuberculous properties (Singh et al., 2021). Luteolin is a Kv1.3 K+ channel inhibitor, profoundly promoting central memory T cells by selectively suppressing effector memory T cells, thereby significantly enhancing the potency of Bacillus Calmette - Guerin vaccine (Singh et al., 2020).

Epigallocatechin Gallate is a drug with broad-spectrum antimicrobial activity. It can directly inhibit early-stage infection by interfering with the attachment of pathogens to host cells, suppressing viral replication, reducing bacterial biofilm formation, and preventing toxin release. Additionally, it can indirectly suppress infection by modulating immune inflammation and exerting antioxidant effects (Zhao et al., 2021). A study reported that green tea and black tea containing epigallocatechin gallate may reduce the risk of tuberculosis infection in the subjects (Maiolini et al., 2020). Epigallocatechin Gallate exhibits a killing effect on Mycobacterium tuberculosis within mouse macrophages through lysosomal acidification and autophagy-induced cellular mechanisms (Sharma et al., 2020).

Beta-sitosterol targets Th1 and Th2 cells, contributing to the normalization of their functions and subsequently enhancing the activity of T lymphocytes and natural killer cells. Additionally, it exhibits inhibitory effects on overactive antibody responses and normalizes cortisol ratios. The reconstitution of these immune parameters is beneficial for numerous disease processes associated with chronic immune-mediated abnormalities, including tuberculosis (Bouic and Lamprecht, 1999). A study has demonstrated that tuberculosis patients treated with Beta-sitosterol exhibited significant improvements in body weight, lymphocyte count, and eosinophil count (Donald et al., 1997).

In summary, the therapeutic impact of GJZLP on tuberculosis exhibits a multifaceted nature, encompassing numerous molecules, targets, and pathways. Its main active components, quercetin, luteolin, epigallocatechin gallate, and beta-sitosterol, act on various targets within the network to produce therapeutic outcomes. These findings suggest that GJZLP has considerable potential for the treatment of tuberculosis and merits further exploration.

Utilizing 118 therapeutic targets, we constructed a protein-protein interaction (PPI) network and subsequently eliminated outlier targets, resulting in 108 targets remaining for a deeper understanding of the core targets. Through the analysis of the PPI network, we identified IL6, TNF, JUN, TP53, IL1B, STAT3, AKT1, RELA, IFNG, and MAPK3 as potential core targets. Among them, IL6, TNF, JUN, TP53, and IL1B are particularly crucial. Previous studies have revealed that, IL-6 and TNF play crucial role in the immune response to tuberculosis (Keane, 2005; Boni et al., 2022). The polymorphisms of IL6 and TNFα are closely linked to the development of tuberculosis, and TNFα serves as a protective factor against pulmonary tuberculosis (Wu et al., 2018). Pre-treatment IL-6 is a biomarker for unfavorable TB treatment outcomes (Gupte et al., 2022). The use of anti-TNF-α agents may cause new tuberculous infections or reactivate latent tuberculous infections (Xie et al., 2014). JUN and TP53 may be involved in the pathogenesis of Mycobacterium tuberculosis infection and invasion by influencing the activity of extracellular domains and protein binding during the infection process (Yuan et al., 2018; Yang et al., 2023). Interleukin-1 (IL-1) is a key participant in the immune response to pathogens, playing a role in promoting inflammation and recruiting immune cells to the site of infection. In tuberculosis (TB), strictly regulating the IL-1 response is crucial to ensure the host’s resistance to infection. Several polymorphisms of the IL-1β gene are associated with increased susceptibility to tuberculosis (Silvério et al., 2021).

To gain a deeper understanding of the biological functions and potential signaling pathways involved in the treatment of tuberculosis with GJZLP, we conducted a comprehensive enrichment analysis of the relevant biological functions and pathways within the Gene Ontology (GO) and the Kyoto Encyclopedia of Genes and Genomes (KEGG). Gene Ontology analysis revealed that the biological processes associated with this treatment primarily encompassed response to molecule of bacterial origin, response to lipopolysaccharide and cellular response to cytokine stimulus. The cellular components involved primarily encompassed vesicle lumen, secretory granule lumen and cytoplasmic vesicle lumen. The molecular functions involved primarily encompassed cytokine receptor binding, cytokine activity, and signaling receptor activator activity. KEGG pathway enrichment analysis revealed that GJZLP treatment for tuberculosis could target multiple signaling pathways, primarily including Lipid metabolism, Atherosclerosis, TNF, IL-17, Toll-like receptor and C-type lectin receptor signaling pathway. The TNF signaling pathway is highly enriched, leading us to speculate that it may be the primary signaling pathway mediating the anti-tuberculosis effects of GJZLP. Relevant studies have demonstrated that the activation of the TNF signaling pathway stimulates the expression of downstream inflammatory factors, thereby triggering a robust inflammatory response within cells. Excessive accumulation of TNF-α in macrophages induces necroptosis, suppressing the survival of Mycobacterium tuberculosis within macrophages (Qian et al., 2022). These results provide a theoretical foundation for further understanding the mechanism of action of GJZLP in the treatment of tuberculosis.

Based on these results, we identified ten key active components and ten core targets. We then performed molecular docking between these core compounds and targets to validate our network pharmacology predictions. The docking results revealed significant binding affinity between the core compounds and targets, with Stigmasterol and AKT1 exhibiting the lowest binding energy and the most stable binding. These findings suggest that the selected core compounds may interact with the targets, thereby influencing the treatment of tuberculosis. These discoveries provide novel insights into the potential molecular mechanisms underlying the therapeutic effects of GJZLP in the treatment of tuberculosis.

GJZLP, as a traditional Chinese medicine formula, has demonstrated promising potential in the treatment of tuberculosis. With the development of precision medicine, GJZLP is expected to facilitate individualized therapy tailored to the specific conditions and genotypes of tuberculosis patients, thereby enhancing treatment outcomes. Elucidating the effective components and their underlying mechanisms of GJZLP is crucial for optimizing its prescription composition and improving its therapeutic efficacy. Furthermore, the synergistic effects of GJZLP in combination with other Western or TCM could enhance the treatment outcomes of tuberculosis. As the internationalization of TCM continues to progress, GJZLP is poised to gain increased recognition from the international medical community, offering more treatment options for patients with tuberculosis.

Subsequently, wet analysis methods can be employed to validate the current research. For instance, quantitative analysis of the active ingredients in GJZLP can be conducted using High-Performance Liquid Chromatography (HPLC), Gas Chromatography (GC), Thin-Layer Chromatography (TLC), spectrophotometry, and chemical analysis. These methods will further corroborate the predictive results of the current analysis, ensuring the quality and efficacy of GJZLP. Cell models can be utilized to investigate the impact of GJZLP on specific signaling pathways such as TNF, IL-17, Toll-like receptor, and C-type lectin receptor. Animal models of tuberculosis can also be established to observe the effects of GJZLP on cell signaling *in vivo*. Additionally, transcriptomics and proteomics techniques can comprehensively analyze the regulatory role of GJZLP on cellular signaling networks. Clinical trials in tuberculosis patients can also be conducted to observe the effects of GJZLP on cell signaling and therapeutic outcomes.

In summary, GJZLP holds significant potential in the treatment of tuberculosis. Its components can be validated through wet analysis methods, and further exploration of its mechanism on cell signaling under different disease conditions can be achieved through a combination of cell experiments, animal studies, omics technologies, and clinical research. This will provide additional scientific evidence to support its clinical utilization. Furthermore, the development potential of Gejie Zhilao Pill extends far beyond the treatment of tuberculosis. Given the widespread application of TCM in the treatment of various diseases, future research can explore the potential of this compound formula in treating other related diseases. Through in-depth studies of its biological mechanisms and modes of action, new applications of Gejie Zhilao Pill in the treatment of other diseases may be discovered.

There are several limitations to this study that need to be taken into consideration. The accuracy and timeliness of data from various databases require scientific validation, and it is possible that the databases may not comprehensively cover all types of TCM and their components. Additionally, compounds or targets that have not been validated or recorded may not have been included in our research analysis. Nevertheless, we recommend leveraging modern bioinformatics techniques for research, as they can provide valuable insights into understanding the mechanisms of TCM. In future studies, researchers can further improve the accuracy and timeliness of database data by implementing measures such as regular updates and quality control.

The bioactive components identified in this study, including quercetin, luteolin, epigallocatechin gallate, beta-sitosterol, formononetin, and stigmasterol do not fully represent the complex composition of GJZLP. These compounds may only contribute to the overall pharmacological effects of GJZLP in the treatment of tuberculosis. Therefore, further pharmacological and molecular experiments are needed to validate our findings. Additionally, the potential active components of GJZLP in the treatment of tuberculosis have not been extensively studied. There is much to be explored regarding the specific roles and mechanisms of these compounds in tuberculosis. Future research should delve deeper into their interactions with identified targets and investigate their impact on tuberculosis-related pathways and processes. Furthermore, studying the synergistic effects of these components in tuberculosis is also worthy of attention. Understanding these interactions could provide valuable insights into the unique pharmacological properties of this formula. Finally, clinical trials are needed to assess the effectiveness and safety of GJZLP in the treatment of tuberculosis. This will help validate its use in real-world settings and provide stronger evidence for its application in tuberculosis management.

## 5 Conclusion

**Figure 9:**
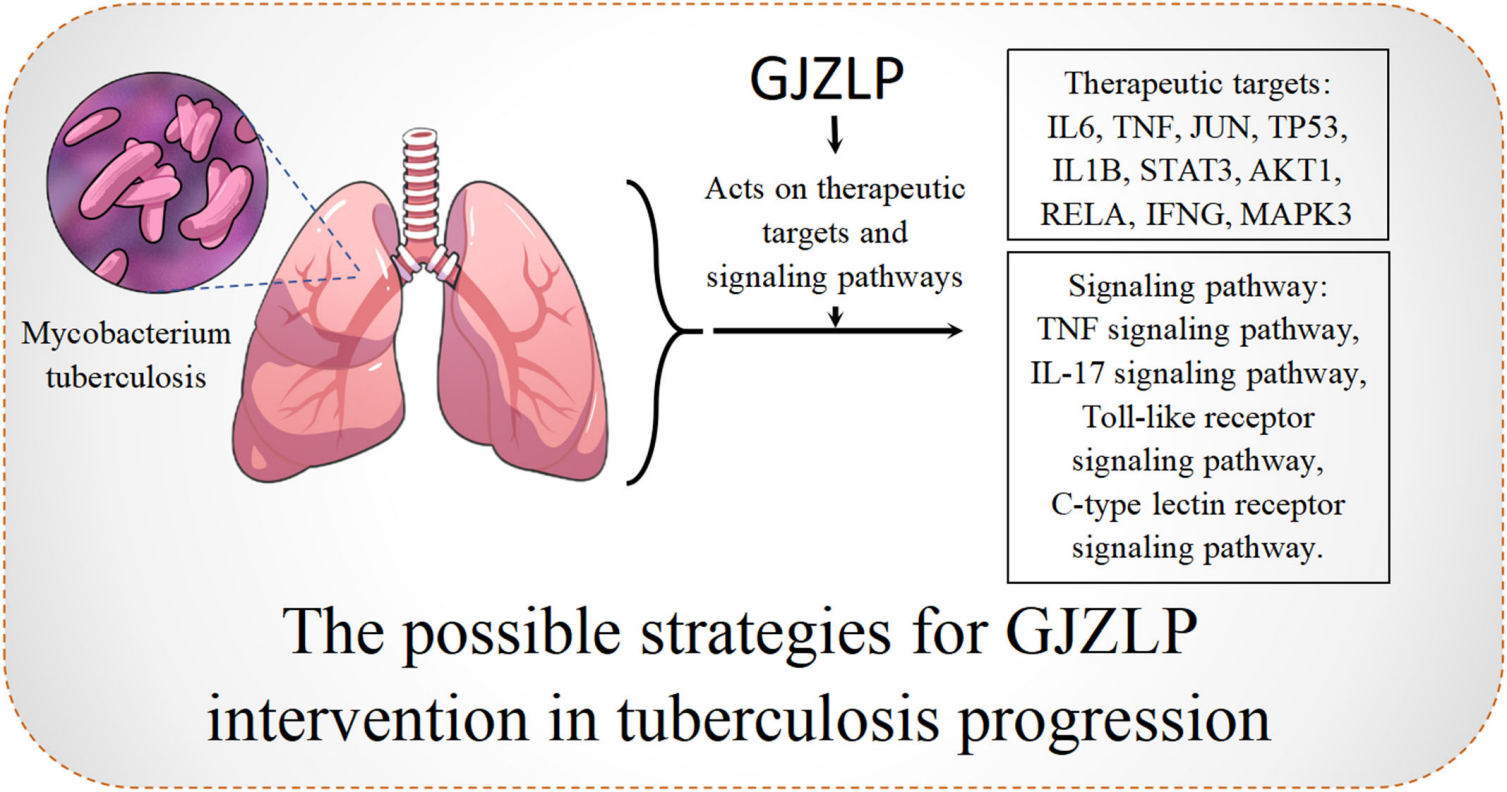
The possible strategies for GJZLP intervention in tubercle progression.

In this research, we employed network pharmacology and molecular docking techniques to initially explore the main chemical components, core targets, and potential mechanisms of GJZLP in the treatment of tuberculosis. Our research indicates that quercetin, luteolin, epigallocatechin gallate and beta-sitosterol may be the primary active components of GJZLP, while protein targets such as IL6, TNF, JUN, TP53 and IL1B are potential targets for GJZLP in treating tuberculosis. These components may play a therapeutic role through TNF, IL-17, Toll-like receptor and C-type lectin receptor signaling pathway. As shown in Prediction of the mechanism (**Figure 9**). These pathways play a role in tuberculosis-associated processes encompassing inflammation, immune response, cell survival and apoptosis.

This study provides a reference for further exploring the mechanism of action of GJZLP in the treatment of pulmonary tuberculosis. Additionally, it offers new insights into the potential therapeutic mechanisms and applications of traditional Chinese medicine formulas, laying a foundation for future antituberculosis drugs development.

## Data availability statement

The original contributions presented in the study are included in the article/**Supplementary Material**. Further inquiries can be directed to the corresponding authors.

## Author contributions

YG: Investigation, Project administration, Writing – original draft. BS: Data curation, Software, Writing – original draft. YH: Investigation, Writing – original draft. WD: Software, Writing – original draft. LW: Conceptualization, Investigation, Writing – review & editing. SS: Funding acquisition, Investigation, Resources, Supervision, Writing – review & editing.
